# Biological Activity of Japanese Quince Extract and Its Interactions with Lipids, Erythrocyte Membrane, and Human Albumin

**DOI:** 10.1007/s00232-016-9877-2

**Published:** 2016-02-10

**Authors:** Paulina Strugała, Sylwia Cyboran-Mikołajczyk, Anna Dudra, Paulina Mizgier, Alicja Z. Kucharska, Teresa Olejniczak, Janina Gabrielska

**Affiliations:** Department of Physics and Biophysics, Wrocław University of Environmental and Life Sciences, C.K. Norwida 25, 50-375 Wrocław, Poland; Department of Fruit, Vegetable and Cereal Technology, Wrocław University of Environmental and Life Sciences, Chełmońskiego 37/41, 51-630 Wrocław, Poland; Department of Chemistry, Wrocław University of Environmental and Life Sciences, C.K. Norwida 25, 50-375 Wrocław, Poland

**Keywords:** Japanese quince, Lipid peroxidation, Erythrocyte and phosphatidylcholine membranes, ^1^H-NMR and fluorometric study, Human serum albumin

## Abstract

The aim of the study was to determine in vitro biological activity of fruit ethanol extract from *Chaenomeles speciosa* (Sweet) Nakai (Japanese quince, JQ) and its important constituents (−)-epicatechin (EC) and chlorogenic acid (CA). The study also investigated the structural changes in phosphatidylcholine (PC) liposomes, dipalmitoylphosphatidylcholine liposomes, and erythrocyte membranes (RBC) induced by the extract. It was found that the extract effectively inhibits oxidation of RBC, induced by 2,2′-azobis (2-amidinopropane) dihydrochloride (AAPH), and PC liposomes, induced by UVB radiation and AAPH. Furthermore, JQ extract to a significant degree inhibited the activity of the enzymes COX-1 and COX-2, involved in inflammatory reactions. The extract has more than 2 times greater activity in relation to COX-2 than COX-1 (selectivity ratio 0.48). JQ extract stimulated growth of the beneficial intestinal bacteria *Lactobacillus casei* and *Lactobacillus plantarum*. In the fluorimetric method by means of the probes Laurdan, DPH and TMA-DPH, and ^1^H-NMR, we examined the structural changes induced by JQ and its EC and CA components. The results show that JQ and its components induce a considerable increase of the packing order of the polar heads of lipids with a slight decrease in mobility of the acyl chains. Lipid membrane rigidification could hinder the diffusion of free radicals, resulting in inhibition of oxidative damage induced by physicochemical agents. JQ extract has the ability to quench the intrinsic fluorescence of human serum albumin through static quenching. This report thus could be of huge significance in the food industry, pharmacology, and clinical medicine.

## Introduction

In the scientific research of recent years, much attention has been paid to the biological activity of natural compounds of plant origin and their potential use in prevention and therapy of several diseases, including civilizational diseases. The Japanese quince (JQ) fruit (genus *Chaenomeles*, family *Rosacea*) is a rich source of polyphenolic compounds, triterpenoids, saccharides, essential oils, and alkaloids (Xianfei et al. 2007). Japanese quince is distributed in Central, East, and Southwest China and is now cultivated worldwide. *Chaenomeles speciosa* has been used in traditional Chinese medicine for thousands of years to treat many diseases, including sunstroke, edema, arthralgia, enteritis, and migraine (Han et al. 2010; Song et al. 2008). Moreover, in recent years, JQ has found application in the treatment of diarrhea, arthritis, and liver ailments (Yao et al. 2013). *Chaenomeles speciosa* was proven to be effective in dopamine transporter (DAT) regulation and antiparkinsonism, as determined by in vitro and in vivo assays (Zhang et al. 2013). Also promising are research results that provide evidence for antitumor activity of JQ. It has been demonstrated that ethanolic extract of *C. speciosa* H22 inhibits tumor growth in mice by direct killing of tumor cells and enhancing immune function (Yao et al. 2013). Other investigators have shown that JQ is an effective inhibitor of the enzymes, MMP-2 and MMP-9, secreted by human leukemia HL-60 cells. Gorlach and co-authors showed that procyanidins isolated from JQ induce apoptosis in human adenocarcinoma cells, whereas Lewandowska and co-authors found a strong antiproliferative effect against breast cancer cells (Gorlach et al. 2011; Lewandowska et al. 2013).

Free radicals present in the body are an integral part of physiology of the living organism (Wong et al. 2005). Their concentration increases under conditions of oxidative stress. Peroxidation of lipids as well as other biomolecules by reactive oxygen species results in disturbances in the structure and function of membranes, which in turn can cause serious diseases, such as atherosclerosis, stroke, cancer, and diseases of the circulatory system (Hendrich 2006). Biological activity of polyphenolic compounds is closely linked to their ability to interact with the bilayer lipid membrane. In the worldwide literature, there were few reports of extensive biophysical studies aiming to explain the molecular mechanisms responsible for protection of the lipid bilayer against peroxidation. The amphiphilic properties of polyphenolic compounds are responsible for their interaction not only with the surface of the lipid membrane (via the polar heads) but also with the deeper areas of the membrane in its hydrophobic part—the acyl chains (Oteiza et al. 2005).

The mechanisms of the therapeutic effects of polyphenolic compounds are not yet sufficiently well understood. One line of research is concerned with both the modulatory impact of these substances on proteins, human serum albumin in particular, and enzymes, including cyclooxygenase and lipoxygenase, and with explaining such modulatory effects.

To determine the potential biological activity of a polyphenolic compound in the body, it is important to know its interaction with bacteria of the intestinal microflora. There are numerous literature data on the activity of polyphenolic compounds in fighting pathogenic bacteria (Tzounis et al. 2008), whereas there are remarkably few studies investigating the influence of polyphenols on the composition and activity of the nonpathogenic gut microbial community.

The aim of the study was to determine in vitro biological activity of fruit ethanol extract from *C. speciosa* (Sweet) Nakai (JQ), and two important components present in JQ, i.e., (−)-epicatechin (EC) and chlorogenic acid (CA), with respect to membranes of phosphatidylcholine liposomes and erythrocyte membranes against peroxidation induced by some physicochemical factors (UVB radiation and AAPH compound). The biophysical studies, using fluorimetric and ^1^H-NMR techniques, were carried out to specify the sites of the interaction between JQ components and the membrane. For the current study, three membrane models were selected: DPPC liposome membranes (one-component membrane to facilitate interpretation of results), PC liposomes of egg phosphatidylcholine (their composition resembling the natural cell bilayer lipid membrane), and red blood cell (RBC) membranes—natural lipid–protein structures. An additional aim of the work was to determine JQ’s ability to inhibit the enzymes (COX-1 and COX-2) involved in inflammatory reactions, to bind to the blood transport protein human serum albumin, and to examine the JQ-mediated increase in the bacteria *Lactobacillus casei* and *L. plantarum* of intestinal microflora. To our knowledge, such comprehensive research on the biological activity of JQ and the proposed potential molecular mechanism of the observed effects/activity has not yet been reported in the scientific literature.

## Materials and Methods

### Materials

DPPH^·^, indomethacin, *N*,*N*,*N*′,*N*′-tetramethyl-*p*-phenylenediamine (TMPD), arachidonic acid from porcine liver, cyclooxygenase 1 from sheep, cyclooxygenase 2 human recombinant, trichloroacetic acid (TCA), 2-thiobarbituric acid (TBA), 1,2-dipalmitoyl-sn-glycero-3-phosphatidylcholine (DPPC), chlorogenic acid, (−)-epicatechin, deuterium oxide (D_2_O), 2,2′-azobis (2-amidinopropane) dihydrochloride (AAPH), and albumin from human serum (lyophilized powder, essentially fatty acid free) were purchased from Sigma–Aldrich (Poznań, Poland). Egg yolk phosphatidylcholine (PC) was obtained from Lipid Products, UK. The probes DPH, DPH-PA, TMA-DPH, and Laurdan were purchased from Molecular Probes (Eugene, Oregon). Tris (hydroxymethyl) aminomethane (Tris:HCl) were obtained from “Chempur” Piekary Śląskie. Bacteria cultures (*L. casei* PCM 2639 and *L. plantarum* PCM 2675) were from the Polish Collection of Microorganisms (PCM, Institute of Immunology and Experimental Therapy, Polish Academy of Science in Wrocław).

### Preparation of Extract

The raw material for the study was Japanese quince [*C. speciosa* (Sweet) Nakai]. The fruit of JQ was collected in the Botanical Garden of Wrocław. It was frozen and then freeze-dried (ChRISTALPhA 1–4 LSC), and just before extraction it was disintegrated with an analytical mill (A11 basic of IKA-Werke, Germany). The process of obtaining the JQ extract was described in detail by Strugała and Gabrielska (2014). In short, fruit lyophilizate was all covered with 70 % (v/v) water–ethanol solution, sonicated, and the alcoholic extract drained. The extract thus obtained was spun in a centrifuge at room temperature and then the ethanol was evaporated to dry weight with a rotary evaporator. The obtained extract was dissolved in distilled water and passed through a column filled with Amberlite resin (XAD4). The column was washed with distilled water until the wash-out of total sugars. Polyphenolic extract was obtained after washing the column with 70 % ethanol. The collected fraction was evaporated in a vacuum evaporator until dry mass. Extract JQ thus obtained was stored at a temperature of −20 °C until assayed.

### Preparation of Erythrocyte Membranes

Erythrocyte membranes were obtained from fresh heparinized pig blood according to the method of Dodge et al. (1963). The content of erythrocyte membranes in the samples was determined on the basis of protein concentration, which was assayed using the Bradford method (Bradford 1976), and it was 100 μg mL^−1^. The choice of pig erythrocytes was prompted by the fact that this cell’s percentage share of lipids is closest to that of the human erythrocyte, and the blood was easily available (Deuticke 1977). Fresh blood was taken each time to a physiological solution of sodium chloride with heparin added.

### High-Performance Liquid Chromatography/Mass Spectrometry (HPLC/MS) Methods

Phenolic compounds were identified by the method described by Kucharska et al. (2015) using the Acquity Ultraperformance Liquid Chromatography (UPLC) system coupled with a quadruple time-of-flight (Q-TOF) MS instrument (Waters Corp., Milford, MA, USA) with an electrospray ionization (ESI) source. Separation was achieved on an Acquity BEH C18 column (100 mm × 2.1 mm i.d., 1.7 μm; Waters). Detection wavelengths were set to 280, 320, 360 nm. The mobile phase was a mixture of 4.5 % formic acid (A) and acetonitrile (B). The gradient program was as follows: initial conditions—99 % (A), 12 min—75 % (A), 12.5 min—100 % (B), 13.5 min—99 % (A). The flow rate was 0.45 mL min^−1^ and the injection volume was 5 μL. The column was operated at 30 °C. UV–Vis absorption spectra were recorded online during HPLC analysis, and the spectral measurements were made in the wavelength range of 200–600 nm, in steps of 2 nm. The major operating parameters for the Q-TOF MS were set as follows: capillary voltage 2.0 kV, cone voltage 40 V, cone gas flow 11 L h^−1^, collision energy 28–30 eV, source temperature 100 °C, desolvation temperature 250 °C, collision gas—argon, desolvation gas (nitrogen) flow rate 600 L h^−1^, data-acquisition range *m/z* 100–1000 Da, and ionization modes—negative and positive. The data were collected using Mass-Lynx V 4.1 software.

Quantification of phenolic compounds was performed by the method described by Sokół-Łętowska et al. (2014) using the Dionex HPLC (Sunnyvale, CA, USA) system equipped with a diode array detector (model Ultimate 3000), a quaternary pump (LPG-3400A), an autosampler (EWPS-3000SI), and a thermostated column compartment (TCC-3000SD), controlled by Chromeleon v.6.8 software. Separation was performed on a Cadenza C5–C18 (75.0 × 4.6 mm, 5 µm) column (Imtakt, Japan) with a guard column. Oven temperature was set to 30 °C. The mobile phase was composed of solvent A (4.5 % formic acid, v/v) and solvent B (acetonitrile). The applied elution conditions were 0–1 min 5 % B, 20 min 25 % B, 21 min 100 % B, 26 min 100 % B, and 27 min 5 % B. The flow rate was 1.0 mL min^−1^, and the injection volume was 20 µL. Flavonols were detected at 360 nm, phenolic acids at 320 nm, and flavanols at 280 nm. Flavonols were quantified as quercetin 3-*O*-glucoside, phenolic acids as 5′-caffeoylquinic acid, and flavanols as (−)-epicatechin. The results were calculated as mg of compound in 1 g dry mass of extract (mg g^−1^ of d.m.) All determinations were performed in duplicate.

### Antioxidant Activity

#### TBARS Test

The procedure was presented previously by Gabrielska et al. (2006b). Lipid peroxidation in phospholipid liposomes was induced by ultraviolet radiation from a bactericidal UVB lamp at 3.5 mW cm^−2^ intensity (UVP radiometer, UK). Lipid peroxidation was measured as the thiobarbituric acid reactive substance (TBARS) level, based on the method of Buege and Aust (1978). TBARS concentrations were estimated using the molar extinction coefficient ε = 156 mM^−1^ cm^−1^. The percentage of PC liposome oxidation inhibition was calculated using the formula:

1 $$\%\, {\text{Inhibition}} = \frac{{C_{0} - C}}{{C_{0} }}\cdot100\,\%$$

where *C*_0_ is concentration of malondialdehyde (MDA) in a sample without antioxidant added (control) and *C* is concentration of MDA in a sample with study compound added, measured at *λ* = 535 nm. All determinations were performed for six independent preparations (*n* = 6) using a Cary 300 Varian spectrophotometer. The IC_50_ parameter was calculated on the basis of plots showing the relation between percentage of lipid oxidation and concentration of the antioxidant. Its value expresses the concentration of an antioxidant that inhibits oxidation by 50 %.

#### Fluorimetric Method

Antioxidant activities of JQ, EC, CA and L(+)-ascorbic acid (AA) were determined using the fluorimetric method described by Cyboran et al. (2015), with minor modifications. The studies were carried out on RBC membranes and PC liposomes, which contained the fluorescent probe DPH-PA. For evaluation of antioxidant activity of substances, the relationship between DPH-PA fluorescence intensity and concentration of free radicals was used. The probe’s fluorescence decreased with its rising oxidation caused by free radicals, supplied by AAPH. Molecules of this compound underwent thermal decomposition into two free radicals each. The value of relative intensity of DPH-PA fluorescence was adopted as a measure of the degree of erythrocyte and lipid membrane oxidation (Arora and Strasburg 1997). It was calculated as a ratio of fluorescence intensity after 30 min of oxidation in the presence of antioxidants to the initial value of the intensity. The polyphenolic compounds of the extract/antioxidant scavenge free radicals and thus cause a lower rate of DPH-PA fluorescence decrease. In this study, we used PC liposomes and RBC membranes (concentration of lipids was 0.1 mg mL^−1^) in phosphate buffer (pH 7.4), incubated for 0.5 h in the dark with the addition of DPH-PA probe at a concentration of 1 µM. The wavelengths of excitation and emission for the probe were as follows: *λ*_ex_ = 364 nm, *λ*_em_ = 430 nm. Oxidation was initiated just before the measurement with AAPH at a concentration of 1 M at 37 °C (control), or in the presence of test substances (JQ, EC, CA, and AA). The concentrations of antioxidants were changed in the range 3–8 µg mL^−1^ for JQ, 1.7–5.0 µg mL^−1^ for EC, 1–3 µg mL^−1^ for CA, and 12–35 µg mL^−1^ for AA. The measurements were conducted with a fluorimeter (Cary Eclipse, Varian). The percentage inhibition of lipid oxidation was calculated on the basis of the following formula:

2 $$\%\, {\text{Inhibition}} = \frac{{(F_{\text{S}} - F_{\text{C}} )}}{{(F_{\text{B}} - F_{\text{C}} )}} \cdot 100\,\%$$

where *F*_S_ is relative fluorescence of the probe oxidized by AAPH in the presence of antioxidant, *F*_C_ is relative fluorescence of control sample oxidized by AAPH without antioxidant, *F*_B_ is relative fluorescence of the blank sample.

### Free-Radical Scavenging Assay

The effect of the extracts on reduction of DPPH^·^ radical concentration was measured spectrophotometrically, as previously described by Brand-Williams et al. (1995). The experiment is described in detail by Strugała and Gabrielska (2014). Reduction of DPPH^·^ in the sample after 15-min incubation with an antioxidant (of fixed concentration) was determined using the formula:

3 $$\%\, {\text{Reduction}} = \frac{{\Delta A_{0} - \Delta A}}{{\Delta A_{0} }} \cdot 100\,\%$$

where Δ*A*_*0*_ is the change of absorbance at *λ* = 517 nm after 15 min in the absence of an antioxidant, and Δ*A* is the change in absorbance at *λ* = 517 nm after 15 min in the presence of an antioxidant. All determinations were performed in six replicates (*n* = 6).

### Packing Order and Fluidity of Membrane

Using the fluorimetric method, the effects of the extracts from the fruit of Japanese quince, (−)-epicatechin, and chlorogenic acid on the physical properties of PC and RBC membranes were examined using the fluorescent probes Laurdan, TMA-DPH and DPH, which become anchored at various depths of the lipid bilayer membrane. The effects of extracts on the packing order of the hydrophilic phases of PC and RBC membrane were examined using the Laurdan probe, while on the basis of changes in fluorescence anisotropies of the probes, DPH and TMA-DPH, the effects of extract on the fluidity of the hydrophobic part and the lipid–water interface of the membrane were examined (Parasassi et al. 1998; Kaiser and London 1998). The prepared PC liposomes and RBC membranes were suspended in a phosphate buffer (pH 7.4), and incubated for 0.5 h in the dark in the presence of a probe. The sample included: PC liposomes or RBC (concentration of lipids was 0.1 mg mL^−1^), fluorescent probe (1 µM) and JQ, EC, CA at a concentration varying within the range of 4–20 µg mL^−1^. Measurements were carried out at room temperature (approx. 20 °C). The excitation and emission wavelengths were as follows: for DPH *λ*_ex_ = 360 nm and *λ*_em_ = 425 nm; and for probe, TMA-DPH *λ*_ex_ = 340 nm and *λ*_em_ = 430 nm. Fluorescence anisotropies for DPH and TMA-DPH were calculated using the formula (Lakowicz et al. 2006):

4 $$A = \frac{{I_{\parallel } - GI_{ \bot } }}{{I_{\parallel } + 2GI_{ \bot } }}$$

where *||*_*I*I_ and *I*_*⊥*_ are the fluorescence intensities observed in directions parallel and perpendicular, respectively, to the polarization plane of the exciting wave. *G* is an apparatus constant dependent on the emission wavelength.

The excitation wavelength for Laurdan was 360 nm, and the emitted fluorescence was recorded at two wavelengths, 440 and 490 nm. Changes in the polar group packing arrangement of the hydrophilic part of the membrane were investigated using the Laurdan probe, on the basis of generalized polarization (GP), and were calculated with the formula (Parasassi et al. 1998):

5 $${\text{GP}} = \frac{{I_{\text{b}} - I_{\text{r}} }}{{I_{\text{b}} + I_{\text{r}} }}$$

where *I*_b_ is fluorescence intensity at *λ* = 440 nm, and *I*_r_ is fluorescence intensity at *λ* = 490 nm. The measurements were conducted with a fluorimeter (Cary Eclipse, Varian). The experiment was performed in six replicates (*n* = 6).

### ^1^H-NMR Measurements

Mixtures of phospholipids (DPPC) with JQ and also epicatechin or chlorogenic acid with JQ were co-dissolved in a chloroform/ethanol mixture (55:1/v:v) at the respective concentration (Hunt and Jawaharlal 1980, Gabrielska and Gruszecki 1996). The lipid concentration in the sample was 36 mM, and in Japanese quince, it was 0.05 mg mL^−1^ (lipid/JQ mixture were 400:1 v/v), and in the case of epicatechin or chlorogenic acid it was 0.36 mM (lipid/epicatechin or lipid/chlorogenic acid mixture were 100:1 v/v). The samples were first evaporated under a stream of nitrogen and then in a vacuum (overnight). Then the samples were hydrated with D_2_O (pH 7.4) and vigorously shaken (15 min) at a temperature above the main phase transition of lipid (41 °C) until optical homogeneity of the mixture was observed. Next the lipid suspension was sonicated with a 20 kHz sonicator (20 kHz, Sonic, Italia) to yield a homogeneous lipid dispersion. Shortly before measurements, a 4 mM praseodymium trichloride (PrCl_3_·6H_2_O) solution was added to the sample with 0.6 mL liposome suspension. ^1^H-NMR spectra were recorded on a Bruker Avance DRX 500 spectrometer (500 MHz-^1^H-NMR). Parameters were as follows: spectral windows 12,019 Hz, digital resolution 0.183 Hz, acquisition, and delay times 2.73 s and 1.00 s, respectively, and acquisition temperature 325 K.

### Cyclooxygenase Activity

The anti-inflammatory activity of the JQ extract was assayed by a spectrophotometric measurement of inhibition of activity of the cyclooxygenases COX-1 and COX-2 accordingly to a modified method given in the work by Jang and Pezzuto (1997). The experiment is described in detail by Strugała et al. (2015). In short, the experimental procedure was as follows: to a cuvette containing Tris–HCl buffer (pH 8.0) the following were successively added: JQ extract (10 µL, in concentrations 40–200 µg mL^−1^), hematin (0.1026 mM) and cyclooxygenases (COX-1 and COX-2) at 1 mg mL^−1^. After mixing and incubation (approx. 3 min), TMPD was added at 24.35 mM. To initiate the reaction, arachidonic acid was added at a concentration of 35 mM. The final volume of the sample was 1 mL. Changes in absorbance of the sample were followed for 3 min by measuring it at 1-min intervals, using a spectrophotometer at a wavelength of 611 nm (Cary 100 Bio Varian) in relation to a reference sample. The measurements were carried out at room temperature. The control sample, instead of the extract, contained the right amount of solvent (10 µL). The experiment was performed in five replicates (*n* = 5).

### Fluorescence Quenching of Human Serum Albumin

Analysis of the potential interaction of JQ extract, EC and CA with human serum albumin (HSA) was performed according to the work by Trnková et al. (2011) with minor modifications. Our method consisted in tracking the quenching of natural HSA fluorescence caused by JQ and its major components (EC and CA) added successively. The final concentrations of JQ varied in the range 6–75 µg mL^−1^, while that of EC and CA was 2–10 µg mL^−1^.

Fluorescence quenching can be described by the Stern–Volmer equation:

6 $$\frac{{F_{ 0} }}{F} = 1 + K_{\text{Q}} \tau_{0} \left[ Q \right] = 1 + K_{\text{SV}} \left[ Q \right]$$

where *F*_0_ and *F* are fluorescence intensities of HSA before and after addition of quencher, respectively, *K*_Q_ is a bimolecular quenching constant, * τ*_0_ is the lifetime of the fluorophore in the absence of quencher, the fluorescence lifetime of a biopolymer is about 5 × 10^−9^ s (Lakowicz 2006), [*Q*] is concentration of the quencher, and *K*_SV_ is the Stern–Volmer quenching constant [*K*_SV_ = *K*_Q_ × * τ*_0_]. This formula was applied to determine *K*_SV_ by a linear regression of the plot of *F*_0_/*F*. All quenching experiments were performed at room temperature for HSA in a phosphate buffer solution of pH 7.4 and final concentration 1.5 × 10^−5^ M and spectra were recorded on a fluorimeter (Cary Eclipse, Varian) equipped with 1.0 cm quartz cells. Fluorescence emission spectra were recorded in the 285–450 nm range with excitation at 280 nm, under continuous stirring. Fluorescence intensity was read at HSA emission maximum of 345 nm. The excitation and emission slits were both set to 5 nm. Fluorescence spectra of JQ extract, CA and EC in buffer were recorded as blanks under the same experimental conditions and subtracted from the corresponding sample to correct the fluorescence background (Papadopoulou et al. 2005). The experiment was performed in three replicates (*n* = 3).

### Effect of Japanese Quince Extract on Growth of Intestinal Bacteria

The effect of the extract on growth of intestinal bacteria was measured spectrophotometrically in liquid cultures in a 96-well plate using microdilution assays. The experiment was conducted according to the procedure described by López-Nicolás et al. (2014) and Duda-Chodak (2012). The working volume in the 96-well plate comprised: 170 μL of appropriate culture medium and 20 μL of cells (1.6 × 10^6^ cells mL^−1^ for *L. plantarum* and 1.9 × 10^6^ cells mL^−1^ for *L. casei*). The JQs were dissolved in 10 μL of 70 % ethanol and studied at a final concentration of 50–250 μg mL^−1^ (the maximum percentage of 70 % ethanol tested was 5 %, which did not affect the growth of any bacteria studied). Bacteria cultures without extract addition constituted the controls. The optical density (OD) of the bacteria cultures was measured after 48 h of incubation at 37 °C using a spectrophotometer (Cary 100 Bio Varian). The number of cells was determined using a Scepter 2.0 (Handheld Automated Cell Counter). The effect of JQ was evaluated by comparing the absorption of the control bacteria to that obtained from culture with extracts. The obtained results were expressed as % of positive control. All measurements were repeated in three 96-well plates with six replicates (*n* = 6).

### Carboxyfluorescein Leakage Studies

The measurements of carboxyfluorescein (CF) efflux were made on the basis of a modified procedure by Yokoyama et al. (2002). PC dissolved in chloroform was evaporated under nitrogen and dried for 120 min in a desiccator. The dry lipid film (5 mg) thus prepared underwent hydration in 0.5 mL Tris–HCl buffer of pH 7.4 with addition of CF at a concentration of 146 mM. In order to facilitate the total dissolution of CF in the process of hydration, 30 µL of 6 M NaOH solution was added. Next, the suspension was sonicated for 30 min. In order to separate the CF trapped in liposomes from free FC, the molecular filtration method was applied, using Sephadex G-50. The column of gel (2 g in 30 mL 1 × 8 cm) balanced with Tris–HCl buffer of pH 7.4 was topped with 0.5 mL solution of liposomes with CF. After the solution was totally absorbed by the gel, the column was eluted with the buffer until the washout of liposomes. The collected liposome fraction, with colorant, was diluted 100 times with the buffer in a 3 mL cuvette. The test sample was then modified with the substances used in appropriate concentrations and incubated for 15 min at room temperature. The final concentrations of JQ, EC and CA varied in the range 4–20 µg mL^−1^. Fluorescence intensity was measured with a Cary Eclipse fluorimeter, at room temperature. Excitation wavelength *λ*_ex_ = 490 nm, and emission wavelength *λ*_em_ = 520 nm. After 15-min incubation the liposome bubbles were destroyed by adding 10 % Triton X-100. The leakage of liposome membranes (L) in % was calculated using the following formula:

7 $$L = \frac{{F_{t} - F_{0} }}{{F_{\infty } - F_{0} }} \cdot 100\,\%$$

where *F*_*t*_ is CF fluorescence intensity in the sample after 15 min, *F*_0_ is initial fluorescence at time = 0 and *F*_*∞*_ is maximal fluorescence of the sample after lysis by Triton X-100. The experiment was performed in three replicates (*n* = 3).

### Statistical Analysis

Data are shown as mean values ± standard deviation (SD). The results were analyzed by one-way ANOVA followed by Duncan test. *P* values <0.05 were considered statistically significant. The program Statistica 12.0 was used for all statistical calculations.

## Results

### Phenolic Content by HPLC/LC–MS Method

By the HPLC/LC–MS chromatographic method we performed quantitative and qualitative analysis of the components present in the extract of JQ. The content of polyphenolic compounds was expressed in mg g^−1^ of preparation (Table 1); it was found to be 349 mg per gram of preparation. The predominant compounds identified in the extract were: procyanidins (which accounted for about 57.8 %), (−)-epicatechin (33 %) and chlorogenic acid (4.4 %).

**Table 1:** Content, mg g^−1^, and characterization of phenolic compounds of the preparation of Japanese quince

Phenolic compounds	Content (mg g^−1^)	*R*_*t*_ (min)	[M − H]^−^ (m/z)	MS/MS fragments (m/z)
Procyanidin B1	3.60	2.29	577	289
(+)-Catechin	2.27	2.69	289	245
Procyanidin trimer	Trace	2.95	865	577/289
Procyanidin B2	115.88	3.41	577	289
(−)-Epicatechin	123.31	4.03	289	245
Procyanidin trimer	47.41	4.39	865	577/289
Procyanidin tetramer	22.17	4.58	1153	577
Procyanidin dimer	12.65	6.48	577	289
Chlorogenic acid	15.64	3.19	353	191
Quercetin-3-*O*-hexoside	1.13	6.65	463	300
Quercetin-3-*O*-ramnoside	5.06	7.78	447	300
Total	349.12			

### Antioxidant and Antiradical Activities

**Fig. 1:**
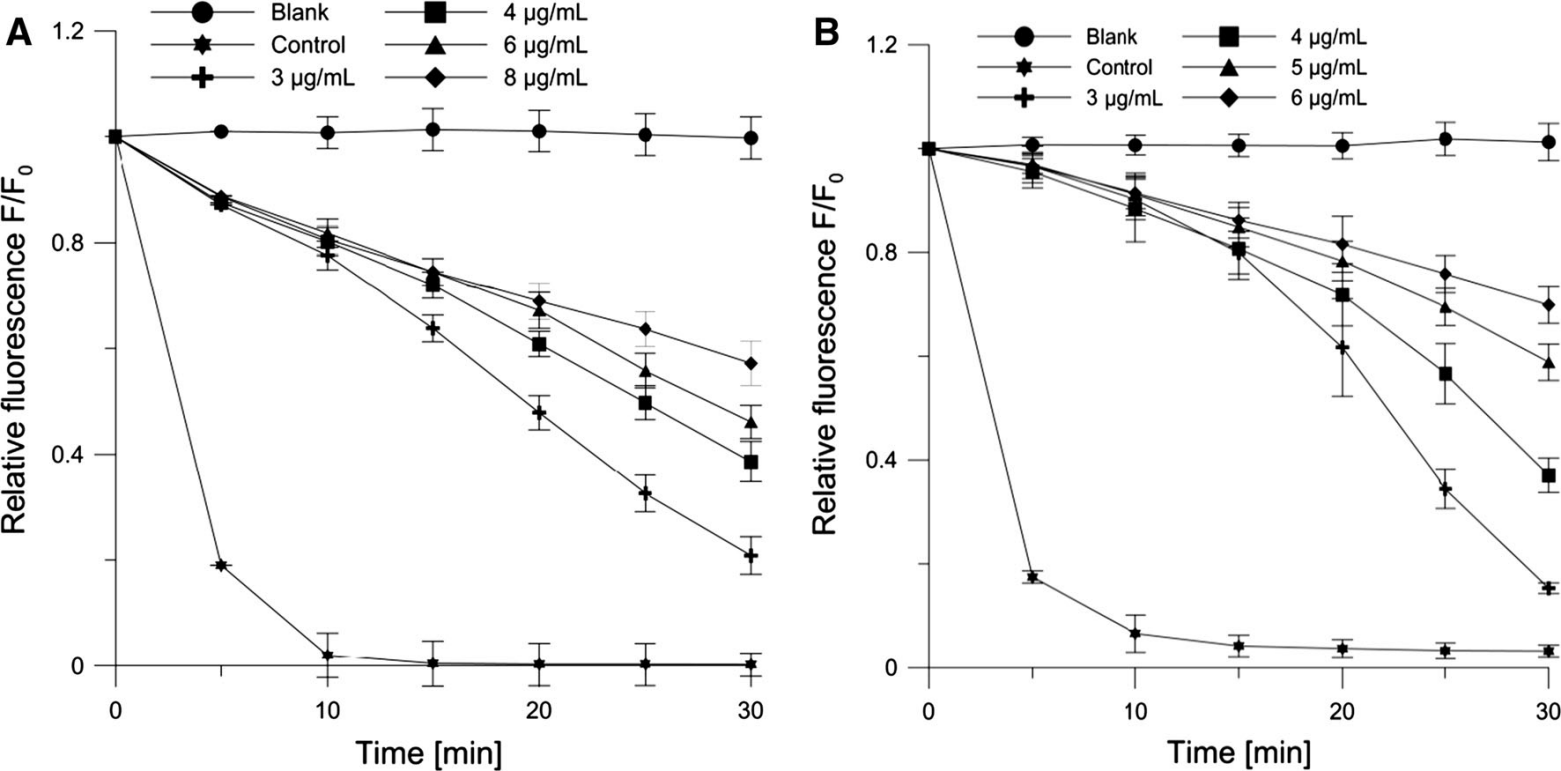
Relative fluorescence intensity of DPH-PA probe as a function of time of oxidation **a** liposomes PC and **b** RBC ghosts for AAPH radicals in the presence of Japanese quince extract at selected concentrations. The relative change in fluorescence intensity *F/F*_0_ is a measure of the degree of lipid peroxidation

Values of the IC_50_ (μg mL^−1^) parameter (the concentration of compounds that caused 50 % inhibition of lipid peroxidation) are shown in Table 2. Using the spectrophotometric method, the antioxidant activity of the substances was assessed on the basis of their ability to inhibit malonic dialdehyde (MDA) emerging during peroxidation of lipids in membranes exposed to UVB radiation; and by the fluorimetric method, the said activity was assessed by the substances’ ability to inhibit oxidation of PC liposomes and RBC ghosts, induced by alkyl radicals produced in thermal disintegration of AAPH. Examples of relative fluorescence intensity kinetic curves of the probe DPH-PA in the presence of JQ extract for PC liposomes and RBC are shown in Fig. 1. With the increasing JQ concentration, the intensity of fluorescence increases in proportion to the degree of lipid membrane oxidation. The kinetic curves of JQ antioxidant action for PC liposomes and model biological RBC membrane are similar. In the first phase of the reaction up to ca. 10 min, JQ protects similarly the membrane lipids against oxidation induced by AAPH in all the used concentrations. Next, when the amount of antioxidant begins to run out (ca.10–30 min), the DPH-PA fluorescence intensity is practically linear.

**Table 2:** Antioxidant (IC_50_) and anti-free radical (EC_50_^DPPH^) (μg mL^−1^) parameters for Japanese quince, (−)-epicatechin, chlorogenic acid, and L(+)-ascorbic acid. Membrane oxidation was induced by AAPH compounds and UVB irradiation. Anti-free radical activity was measured in the DPPH^·^ test

Inducer/membrane/DPPH	Japanese quince (μg mL^−1^)	(−)-Epicatechin (μg mL^−1^)	Chlorogenic acid (μg mL^−1^)	L(+) ascorbic acid (μg mL^−1^)
AAPH				
PC	6.48 ± 0.91	2.48 ± 0.29	2.45 ± 0.28	22.80 ± 2.19
RBC	4.81 ± 0.17	2.95 ± 0.17	0.90 ± 0.08	20.10 ± 1.15^a^
UVB				
PC	20.12 ± 0.88	5.36 ± 0.75	6.69 ± 0.71	115.3 ± 2.5
DPPH				
	4.14 ± 0.05	3.48 ± 0.01	7.34 ± 0.03	3.26 ± 0.001

*PC* liposomes of phosphatidylcholine, *RBC* erythrocyte ghosts

^a^Cyboran et al. (2015)

Based on the plots of oxidation kinetics, the percentage of oxidation inhibition after 30 min was calculated for the extract JQ, EC and CA. The results of antioxidant tests show that substances in varying degrees protect membrane lipids from oxidation induced by physicochemical factors. Antioxidant activities of the extract and its major components depend on the applied inducer of free radicals (UVB radiation or AAPH compound) and the type of membrane. The results of the study show that all tested compounds protect the lipid membrane against free radicals, though more efficiently against those induced by AAPH than by UVB radiation (IC_50_ in the case of UVB is about 3–11 times greater than in the case of AAPH). For EC and CA, the differences in value of IC_50_ for both the oxidizing agents were relatively small. IC_50_ values of studied substances are compared with those of L (+)-ascorbic acid, which showed weaker activity than JQ, by about 3.5 times for the fluorimetric method and 5.7 times for the spectroscopic method.

The results for antiradical activity with respect to the free-radical DPPH^·^ are presented in Table 2. The calculated parameter EC_50_ varied according to the following sequence: L (+)-ascorbic acid = (−)-epicatechin < Japanese quince < chlorogenic acid. The test results indicate that JQ has about 1.7 times higher antiradical activity than CA and only about 1.3 times lower than the ability of *L* (+)-ascorbic acid to scavenge DPPH radicals.

### Packing Order and Fluidity of Membrane

**Fig. 2:**
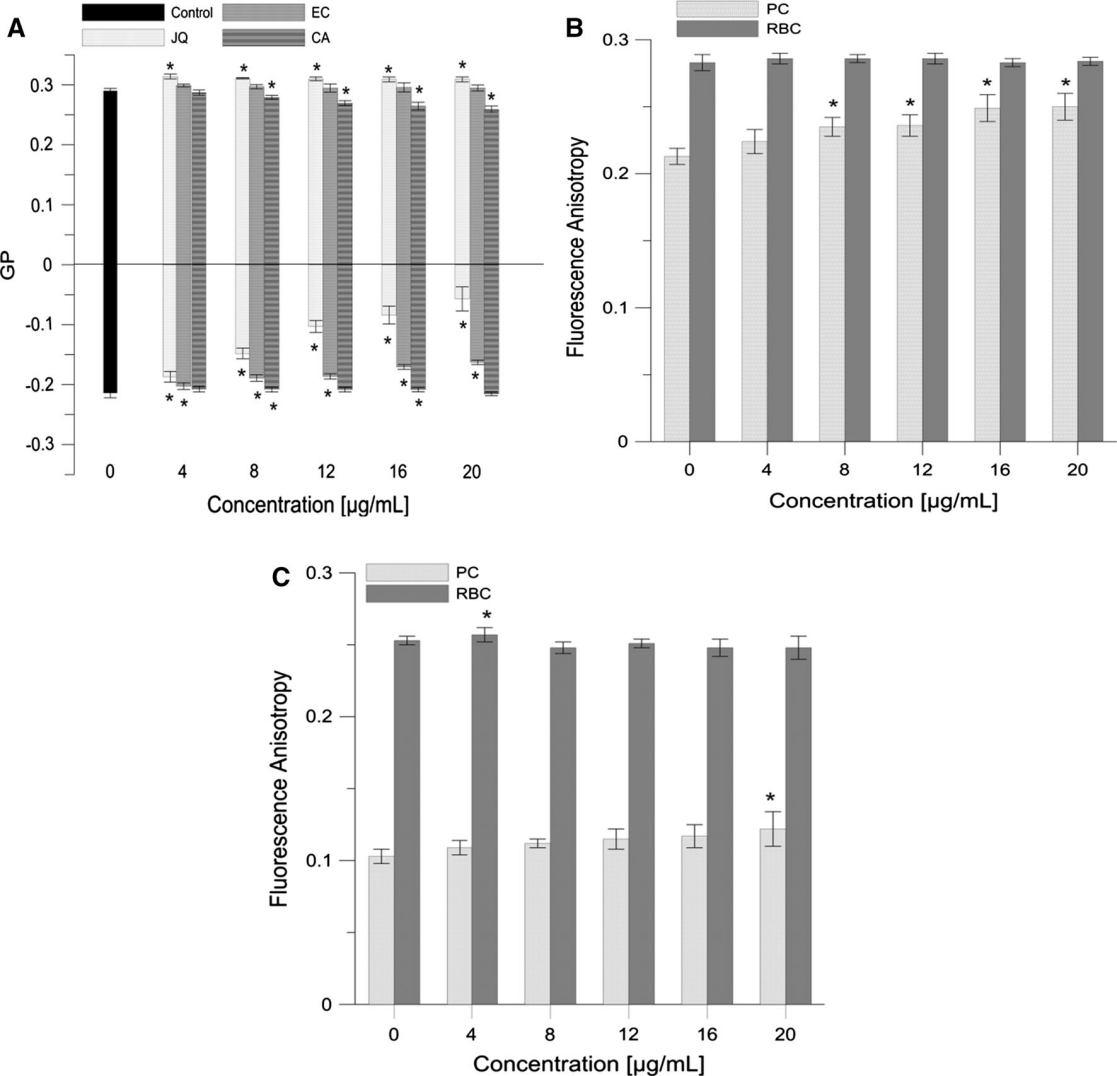
**a** Generalized polarization (GP) of Laurdan probe as a function of Japanese quince (JQ), (−)-epicatechin (EC) and chlorogenic acid (CA) concentration (positive values for RBC, negative values for PC); **b**, **c** TMA-DPH and DPH probes fluorescence anisotropy as a function of concentration of Japanese quince. PC—phosphatidylcholine liposomes, RBC—erythrocyte membrane (ghosts). Values are mean ± SEM, *n* = 6. Means labeled with *asterisk* (*) are significantly (*p* < 0.05) different from control

Using the fluorescent probes Laurdan, TMA-DPH and DPH located at various depths of the bilayer lipid membrane, we studied the effects of JQ extract and its selected components, EC and CA, on the properties of the hydrophilic and hydrophobic regions of the model membrane created from PC lipids and also RBC membrane to assess the depth at which JQ molecules reside in the membrane. The Laurdan probe, chromophore of which is at the level of the backbone of glycerol molecules, informs us about the impact of the extract in the hydrophilic membrane region (Parassasi et al. 1998). The study showed that the extract, in the concentration range of 4–20 μg mL^−1^, caused a statistically significant (*P* < 0.05) increase in the value of the GP (negative values) for the one-component PC membrane, while in the case of the multicomponent membrane of erythrocytes, the increase of GP (positive values) was small compared to the control sample (Fig. 2). EC used in the same concentration range, as well as JQ extract, also caused a statistically significant (*P* < 0.05) increase in the GP parameter in the case of PC membrane and a slight increase in RBC membranes. CA, in the case of both PC and RBC membranes, caused a slight drop in the GP parameter (not exceeding 10 % relative to the control). On the basis of changes in the value of the DPH and TMA-DPH probes’ fluorescence anisotropy, the effects of the extract on fluidities of the hydrophobic area and the lipid– water interface of the liposome membrane were determined. The results of the measurements have shown that JQ extract slightly increases fluorescence anisotropy of both the probes in the case of PC membrane (*P* < 0.05), whereas in the case of RBC membranes, no changes in fluorescence anisotropy can be noted (Fig. 2b, c) and hence, JQ extract in the applied concentration of 20 μg mL^−1^ caused an approximately 18 % increase in the anisotropies of both the probes, TMA-DPH and DPH, localized in PC membrane compared to the control sample. The analysis of the results for EC allows us to conclude that this compound causes an increase in anisotropies of both the probes, TMA-DPH and DPH, anchored in a PC membrane, although to an extent not exceeding 5 %. It does not, however, induce changes in that parameter in the close microenvironment of the probes in the RBC membrane. The effect of CA on the microenvironment of the fluorescent probes concerned only the probe TMA-DPH present in the PC membrane. It was about an 8 % increase in anisotropy at 20 μg mL^−1^ concentration.

### ^1^H-NMR Studies

**Fig. 3:**
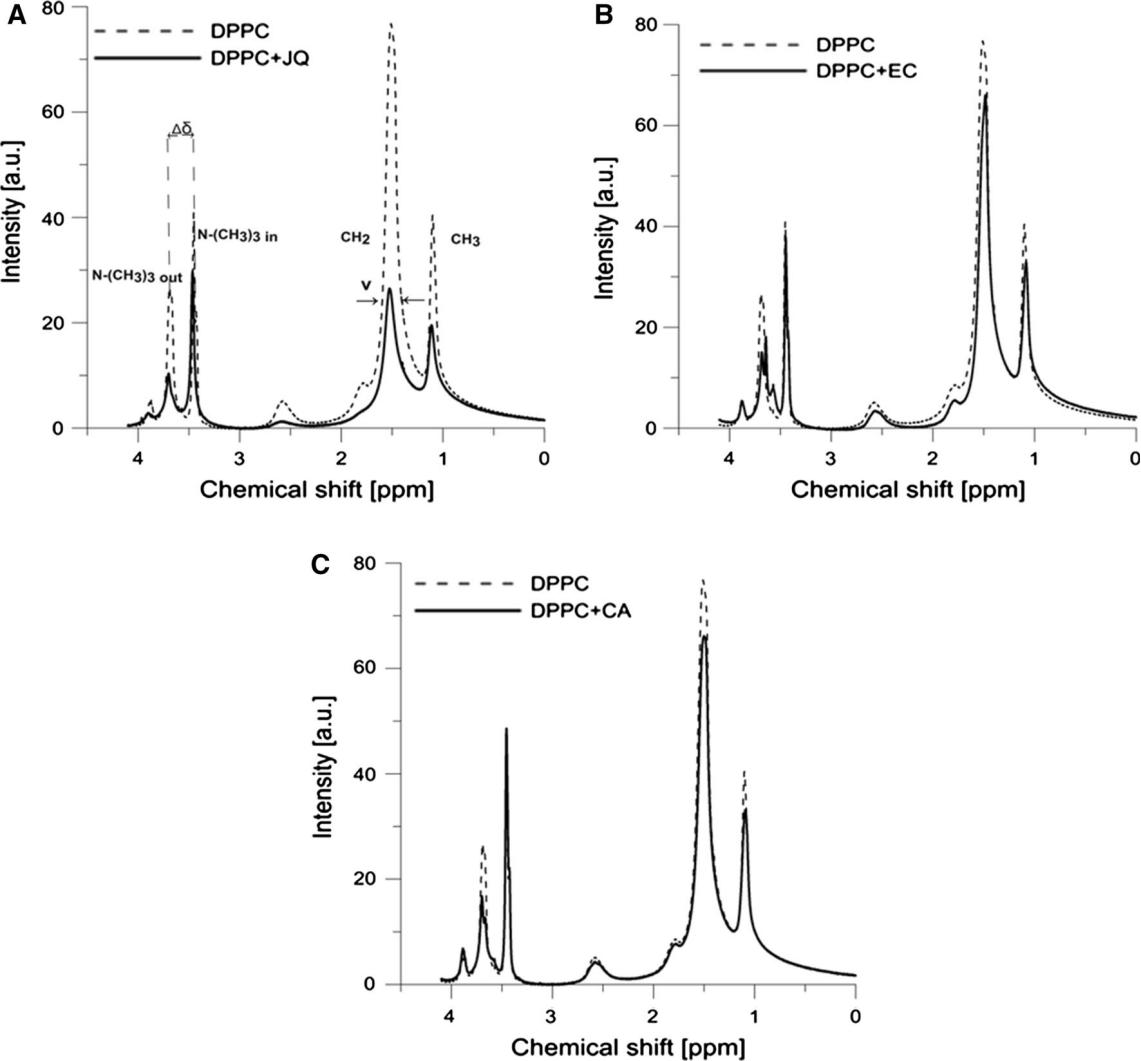
^1^H-NMR spectra of liposomes formed from pure DPPC and DPPC with **a** Japanese quince, **b** (−)-epicatechin (EC); **c** chlorogenic acid (CA), at 1 mol%. PrCl_3_ (4 mM) was added to the samples before measurement

Figure 5 presents ^1^H-NMR spectra of DPPC liposomes and treated with JQ extract (0.05 mg mL^−1^) (400:1 v/v chloroform/ethanol) (Fig. 3a), 1 mol% EC (Fig. 3b), and 1 mol% CA (Fig. 3c). Several bands are visible in the spectra that correspond to the following major molecular features of DPPC membranes: –CH_3_ and –CH_2_ groups of the hydrophobic region of the membrane, as well as the bands of –N^+^–(CH_3_)_3_ from the polar head region of the membrane. Addition of praseodymium ions results in a split of the ^1^H-NMR band (Δ*δ*) corresponding to the ammonium group, –N^+^–(CH_3_)_3_, owing to the pseudo-contact shifts produced by the shift reagents from the group of lanthanides (e.g., Pr^3+^). The resonance maximum shifted toward higher ppm values corresponds therefore to the lipid molecules forming the outer leaflets of the liposome membranes [–N^+^–(CH_3_)_3Out_], whereas the one shifted toward lower ppm values corresponds to the inner liposome surface [–N^+^–(CH_3_)_3In_]. The ratio of the areas under the signal assigned to the outer layer to that assigned to the inner layer (*I*_Out_/*I*_In_, outer to inner) is proportional to the number of the choline heads (molecules) in the outer and inner layers. It is obvious that the number of lipid molecules in the outer layer is greater than that in the inner layer, so for unilamellar liposomes the ratio *I*_Out_/*I*_In_ is >1. Addition of JQ extract or its components such as EC or CA causes a change in the spectra parameters (Table 3): in the full width at half height (*ν*) of the ^1^H-NMR bands, in the ratio *I*_Out_/*I*_In_ and, in some case, in the splitting (Δ*δ*) of the band corresponding to the ammonium group –N^+^–(CH_3_)_3_, from the outer and inner leaflets of the membrane. Addition of JQ caused the increase in the full width at half height (ν) of the ^1^H-NMR bands. A slight increase of 2 % in the case of the –CH_2_ group was observed. The presence of JQ components caused a strong increase in *ν*, by 56 and 45 %, of the outer and inner leaflets of the membrane, respectively. Moreover, presence of JQ dramatically changed the *I*_Out_/*I*_In_ ratio, from 1.250 in pure DPPC to 0.5649 in liposomes with addition of the examined extract (Table 3). A relatively high ordering effect in the hydrophilic region (restriction of motional freedom) of membrane is observed in the presence of EC. Addition of EC causes a relatively large increase in ν, by 24 and 13 %, of the outer and inner leaflets of membrane, respectively, whereas CA induced an increase in ν only by 18 and 5 %. A small decrease of 5 or 2 % in the case of –CH_2_ group was observed in the presence of EC or CA, respectively. At the same time an increase in the full width at half height (*ν*) the ^1^H-NMR band in the case of the terminal methyl group was observed in the presence of all modifiers (studied extract and polyphenols). EC addition also results in a 10 % decrease (Δ*δ*) in the splitting parameter of the resonance maximum corresponding to polar head groups, whereas the extract only slightly (3 %) and CA does not change this parameter. Presence of CA molecules in the DPPC membrane caused a marked decrease, by about 44 %, in the *I*_Out_/*I*_In_ ratio, from 1.2500 in pure DPPC to 0.8696, while EC molecules change this ratio only by 5 %, increasing the value to 1.3070.

**Table 3:** Parameters of ^1^H-NMR spectra at 325 K of DPPC liposomes and DPPC liposomes with the addition of Japanese quince (JQ) extract (v/v 400:1) and DPPC with addition of 1 mol% chlorogenic acid (CA) and (−)-epicatechin (EC)

Liposome composition	Parameter					
	ν –N^+^–(CH_3_)_3Out_ (ppm)	ν –N^+^–(CH_3_)_3In_ (ppm)	Δδ (ppm)	ν –CH_2_ (ppm)	ν –CH_3_ (ppm)	*I*_Out_/*I*_In_
DPPC	0.0644	0.0268	0.2420	0.1300	0.0715	1.2500
DPPC + JQ	0.1007	0.0390	0.2360	0.1328	0.1134	0.5649
DPPC + EC	0.0801	0.0305	0.2180	0.1230	0.0823	1.3070
DPPC + CA	0.0760	0.0282	0.2420	0.1268	0.0851	0.8696

### Cyclooxygenase Inhibition

The results for inhibition of enzymes involved in the inflammatory reactions of the body are expressed as the IC_50_ parameter, as shown in Table 4. They show that the JQ extract is about 2 times more efficient in COX-2 inhibition (IC_50_ = 74.12 µg mL^−1^) than COX-1 (IC_50_ = 150.44 µg mL^−1^). The results are compared to a synthetic anti-inflammatory drug—indomethacin.

**Table 4:** Values of IC_50_ for the Japanese quince extract and indometacin, i.e., concentration at which 50 % inhibition of the activity of cyclooxygenase-1 (COX-1) and cyclooxygenase-2 (COX-2) occurs

Compound	IC_50_ (µg mL^−1^)	
	COX-1	COX-2
Japanese quince	150.44 ± 11.15	72.10 ± 8.10
Indometacin	9.15 ± 0.23	7.60 ± 0.68

### Fluorescence Quenching of Human Serum Albumin

**Fig. 4:**
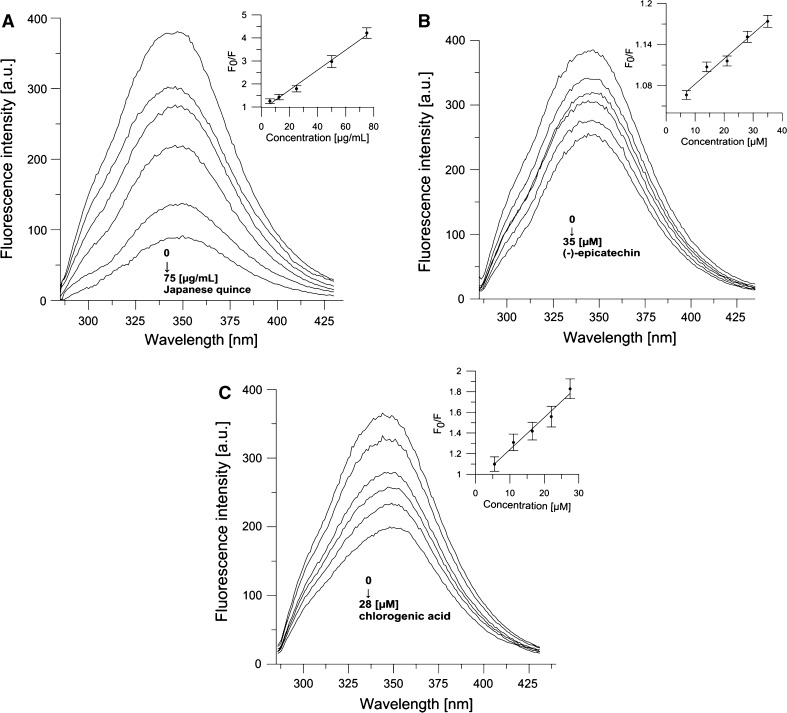
Emission spectra of HSA in the presence of various concentrations of Japanese quince (JQ) (**a**), (−)-epicatechin—EC (**b**), chlorogenic acid—CA (**c**), and Stern–Volmer plots of *F*_o_/*F* against concentration for JQ, EC and CA. (HSA = 1.5 × 10^−5^ M, *λ*_ex_ = 280 nm, *T* = 295 K)

Quenching of protein intrinsic fluorescence was employed for a more detailed study of the JQ, EC and CA interaction with HSA. Fluorescence intensities were read at an emission wavelength of 340 nm where the emission maximum of HSA was located. Based on emission spectra of the albumin, excited by radiation of *λ*_max_ = 280 nm attributed to tryptophan and tyrosine residues, a lowering of albumin fluorescence intensity was observed caused by the studied compounds, when their concentration increased (Fig. 4a, b, c). These results suggest that JQ extract can interact with HSA, causing quenching of its fluorescence. In order to clarify the fluorescence quenching mechanism (induced by JQ and its compounds), the fluorescence quenching data were analyzed using the Stern–Volmer equation. On the basis of Eq. 6, binding constants (*K*_SV_) for the ligand–protein complex were determined using the linear regressions of the plots of *F*_0_/*F* versus [*Q*]. The plots were linear in the following ranges of concentration for all tested compounds: JQ 6-75 µg mL^−1^, CA 5–28 × 10^−6^ M (2–10 µg mL^−1^), EC 7 × 10^−6^ M (2–10 µg mL^−1^ (Fig. 4). For JQ extract, the binding constant *K*_SV_ = 43.4 × 10^3^ mL g^−1^, for EC and CA *K*_SV_ = 3.7 × 10^3^ M^−1^ and *K*_SV_ = 30.2 × 10^3^ M^−1^, respectively.

### Effect of Japanese Quince Extract on Intestinal Bacteria

**Fig. 5:**
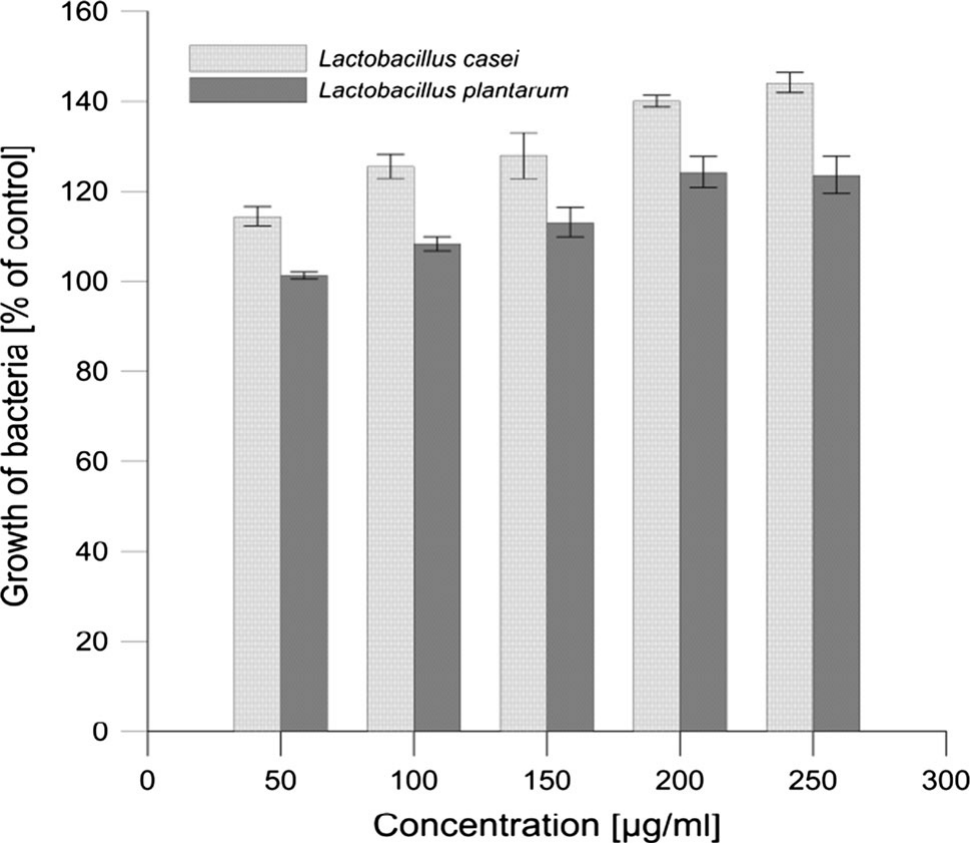
Effect of Japanese quince extract (50–250 µg mL^−1^) on the growth of probiotic bacteria (*Lactobacillus casei* and *Lactobacillus plantarum*). Data are percentages compared with control (ethanol–water solution in culture medium)

The stimulatory effect of JQ extract on the beneficial *Lactobacillus* strains was evaluated in liquid cultures, and their optical densities (OD) were measured. The effect of extract on growth of the intestinal bacteria *L. casei* and *L. plantarum* is presented in Fig. 5. Results are shown the growth (% compared to control) of bacteria in the presence of JQ extract. Ethanol (70 % solution) showed no significant effect on bacterial growth at the assayed concentrations and was used as a control. As one can notice, JQ extract positively affects the growth of intestinal bacteria in the concentration range 50–250 µg mL^−1^. In the case of the bacteria *L. casei*, the growth increase was 14–44 % relative to the control, and in the case of *L. plantarum* the extract stimulated growth by 1–23 %.

### Carboxyfluorescein Leakage Studies

**Fig. 6:**
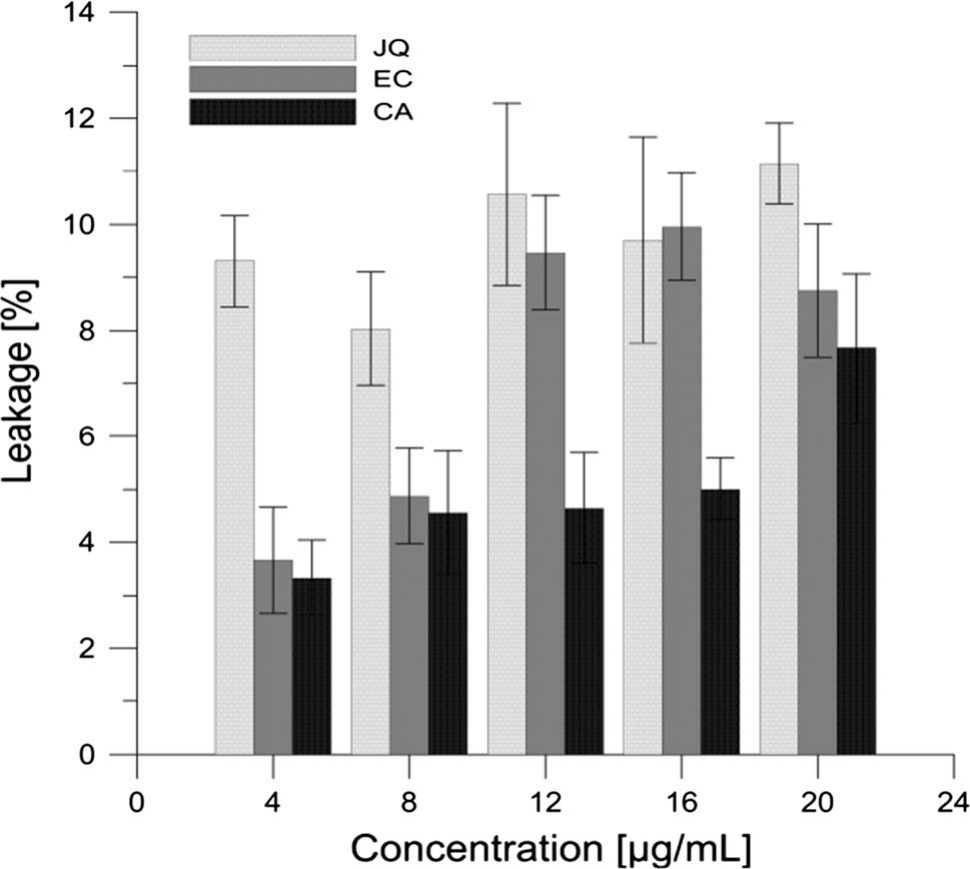
Extent of leakage of carboxyfluorescein from PC liposomes expressed as a percentage after 15-min incubation, as a function of concentration, for Japanese quince (JQ), (−)-epicatechin (EC) and chlorogenic acid (CA)

We investigated the leakages of the fluorescent probe CF from inside of PC liposomes in the control probe and probes modified with JQ extract, EC, and CA. CF fluorescence can be detected only after the probe is released from the liposomes. All the compounds studied were tested in the same concentration range (from 4 up to 20 μg mL^−1^). Their influence on liposome permeability is shown in Fig. 6, which shows the percentage of CF efflux from liposomes as a function of the concentration of the test compound. The results show that the JQ extract in the whole range of used concentrations (4–20 µg mL^−1^) induced a similar degree (9–11 %) of the CF release from inner part of PC liposomes. (−)-Epicatechin at concentrations of 4–8 µg mL^−1^ causes a slight leakage of CF (3.6–4.8 %), while at concentrations of 12–20 µg mL^−1^, it is about 9 %. Chlorogenic acid causes a slight CF leakage from the liposomes, (approx. 8 %), but only at the concentration of 20 µg mL^−1^.

## Discussion

The fruit of JQ is a rich source of a variety of compounds, including phenolics, with an original and unique composition responsible for its biological properties. Currently, the interest of researchers in natural substances with health-promoting properties is increasing.

With the LC–MS and HPLC method, in the JQ extract we identified approximately 35 % of phenolic compounds belonging to 3 groups: flavan-3-ols (93.7 %) (dominated by (−)-epicatechin and procyanidin B2), phenolic acid, and flavonols (Table 1). The results seem to be in good accordance with those presented by Du and co-workers, who identified five representative compounds (chlorogenic acid, (+)-catechin, (−)-epicatechin, and procyanidin B1, procyanidin B2), in five species of *Chaenomeles* (Du et al. 2013). In this study, total flavan-3-ols content [including (+)-catechin, (−)-epicatechin and procyanidin oligomers] accounts for 94–99 % of the total polyphenol content.

The studies of antioxidant activity of JQ extract with respect to the biological membrane (erythrocytes) and lipid (egg PC) membrane showed that the extract protects membrane lipids, to varying degrees, before oxidation induced by physicochemical factors. It turned out that JQ more effectively protects the lipid membrane against free radicals induced by AAPH than those produced by UVB radiation. This may be due to the fact that free radicals produced by AAPH in an aqueous environment are better eliminated by the hydrophilic components of the extract, which are incorporated in the lipid membrane in the immediate vicinity of the radicals. UVB radiation, penetrating the entire membrane, can generate free radicals in its hydrophobic interior, where fewer JQ components penetrate. The results of the biophysical studies obtained by means of fluorescent probes, including Laurdan, suggest that the distribution of JQ components in the membrane hydrophilic region at the interphase boundary is the dominant factor conducive to effective scavenging of AAPH^·^ radicals that attack the membrane from the side of the aqueous medium. It should also be taken into account that antioxidant capacities of JQ components depend on reactivities of the free radicals generated by the compound AAPH or UVB radiation (superoxide anion, hydroxyl radicals, hydrogen peroxide, and others) (Heck et al. 2004; Gülçin 2012). The results of subsequent studies have shown that two important components of the extract, EC and CA, protect the membranes of RBCs and lipid membranes against the peroxidation process to a higher degree than JQ (Table 2). EC and CA have shown from around 38 to 73 and from 62 to 81 %, respectively, higher antioxidant activities than JQ extract. The results obtained suggest that the high antioxidant activity of JQ extract is probably largely due to its main phenolic components. Antioxidant capacity of powdered *C. speciosa* fruit in vivo was confirmed by other researchers who showed that it may reduce the serum levels of low-density lipoprotein cholesterol and total cholesterol, increase glutathione peroxidase activity, and decrease the relative atherosclerotic plaque area of the aortic sinus and aortic arch in mice (Tang et al. 2010).

Determination of the antiradical activity of JQ extract was conducted using the stable model radical DPPH^·^. Studies have shown that the extract effectively scavenges DPPH^·^ radicals (EC_50_ = 4.18 µg mL^−1^) (Strugała and Gabrielska 2014). EC and ascorbic acid showed slightly higher antiradical activity than the extract tested, at variance with the properties shown by CA, which was approximately 1.7-fold less effective in leveling the radical DPPH^·^. A similar relationship was found using the DPPH^·^ test for antiradical capacity of methanolic apple extracts, whose polyphenolic composition [rich in procyanidin B2, (−)-epicatechin and chlorogenic acid] was quantitatively comparable to that of JQ extract (Panzella et al. 2013). This paper concluded that procyanidins were the major determinant of the antioxidant activity while chlorogenic acid contributed to a lesser extent.

An important biological property of plant extracts is their ability to interact with the lipid bilayer. In the literature so far, there have been no reports on the interaction of JQ with biological membranes; therefore, the research carried out in this direction is of pioneering character. The ability to interact with lipid membranes is closely coupled with extract components’ nonpolar properties. ^1^H-NMR technique was proved to be a good tool for investigation of the dynamics and structural properties of the membrane. The analysis of the full width at half height (*v*) of certain maxima in ^1^H-NMR spectra recorded from liposomes with extract added indicated that extract components are localized within the lipid bilayer. The effect of the broadening of spectral peaks which is directly related to restriction in segmental movement of the lipid heads was strong in the case of –N^+^–(CH_3_)_3_ groups in the outer and inner leaflets of the liposome membrane. This is an indication of a strong ordering effect by the extract molecules on the hydrophilic parts the lipid bilayer. The effect of broadening of spectral peaks, directly related to limitations in the segmental movement of lipid molecules, was slight in the case of –CH_2_ groups. Additionally, the effect of multilamellar liposome formation in the presence of extract was observed (Table 3, *I*_Out_/*I*_In_ = 0.5649) and compared with small unilamellar liposomes formed in the case of pure DPPC lipids (*I*_Out_/*I*_In_ = 1.2500). The smaller the unilamellar liposomes, the lower is the number of lipid molecules that can fit in the inner layer of the liposome and the higher is the ratio *I*_Out_/*I*_In_ (Gabrielska and Gruszecki 1996). As can be seen from the data (Δ*δ* decreased by about 3 %), the inclusion of extract components in the membrane slightly reduced the penetration of Pr^3+^ cations into the head region of the membrane. The comparison of proton resonance NMR spectra for the choline groups of membrane with extract added with membrane with phenols added, i.e., EC and CA, revealed a significant decrease in the motional freedom of polar head groups (EC > CA). The results also indicate slight fluidization of the hydrophobic core of the membrane in the presence of EC and of CA. (−)-Epicatechin, but not chlorogenic acid, after incorporation into DPPC membrane, significantly reduces the penetration of Pr^3+^ to the polar region of the membrane. In contrast, the presence of CA but not EC during formation of the liposome membrane induced the formation of the multilamellar liposome structure. The finding from our investigation is that JQ components, including EC and CA, are incorporated into the DPPC membrane. In line with our data, Sinha and co-authors showed that quercetin, a compound with 5 hydroxyl groups in its molecular structure, i.e., with the same number of OH groups as the (−)-epicatechin molecule, is located at the lipid/water interfacial region (Sinha et al. 2012). The localization and distribution of different flavonoid molecules in POPC membrane were studied using nuclear magnetic resonance spectroscopy by Scheidt et al. (2004). The authors of that work also suggest that distribution of flavonoids (flavone, chrysin, luteolin, and myricetin) in the membrane is closely related to their polarity. These results confirmed the conclusions of previous reports (Lehtonen et al. 1996; Hendrich et al. 2002; Gabrielska et al. 2006a, b). These authors concluded that flavonoids could reach all regions of the bilayer, and thus could protect the whole bilayer from oxidation. There have been a few studies in which ^1^H-NMR and other physical methods were used to investigate the changes in dynamic and structural properties of lipid membranes as a result of interactions with flavonoids and other phenols (Pawlikowska-Pawlęga et al. 2014; Wesołowska et al. 2009). It can be concluded that such a membrane-stabilizing effect might contribute to the antioxidative properties of flavonoids, including phenolic compounds. The results of those authors agree well with our results obtained using fluorescence probes.

The results obtained by means of fluorescent probes that become imbedded at different depths of the lipid bilayer have shown that JQ extract modulates both the hydrophilic and the hydrophobic regions of the PC lipid membrane and slightly affects the RBC membrane. Changes in the polar region induced by JQ signify a strong ordering action of extract components in the area around Laurdan probe molecules, resulting in increased general fluorescence polarization. It is known from the literature that in lipid membranes the Laurdan probe is sensitive to the amount of water molecules present within the bilayer. If the lipids are well ordered, water molecules will have less access to the Laurdan probes embedded in the membrane, thus resulting in a high value of GP (Sanchez et al. 2007). On the basis of obtained increases in fluorescence anisotropy from the hydrophobic region of PC membrane (Fig. 2b, c), one can expect that JQ extract will impose a slight restriction on the dynamics of acyl chains of the lipid bilayer, without affecting the RBC membrane dynamics (Fig. 2b, c). Tammela and co-workers ascribe the increase in TMA-DPH and DPH probes’ anisotropy to a decrease of fluidity in the probes’ vicinity and thus stiffening of the lipid molecules or their segments (Tammela et al. 2004). The lack of impact of JQ on the RBC membrane could be the result of different construction of this membrane in comparison to model membranes of liposomes. PC liposomes are formed from phospholipids of the same structures of the head groups and acyl chains, in varying lengths and degrees of bonds unsaturation, which entails great mobility and thus low structural order. In contrast, the erythrocyte membrane, due to the presence of sphingomyelin, cholesterol or the possible presence of cytoskeleton proteins, has a more ordered structure in contrast to the liquid, disordered structures of the PC liposomes (Bernhardt and Ellory 2003). Different effects of the extract from the fruit of the apple on changes in fluidities of the model liposome membrane and erythrocyte membrane have been reported (Bonarska-Kujawa et al. 2011). In our study, it was found that the extract caused a decrease of the GP parameter in the case of erythrocyte membrane, whereas there was an increase in GP, i.e., stiffening of the membranes formed of egg lecithin. Test results also indicate that the increase in the order of lipid heads and the slight decline in the fluidity of the hydrophobic PC membrane core are mostly caused by EC and not by CA (Fig. 2). The interaction of CA with the polar heads of lipids in erythrocyte membranes resulting in reduction of the packing is in accordance with the relationship presented previously (Bonarska-Kujawa et al. 2015; Cyboran et al. 2013). Studies of the interactions of JQ extract and its components with the membrane composed of different lipids suggested that the interaction may rely on the establishment of hydrogen bonds between the hydroxyl groups of the flavonoid and the polar headgroups of phospholipids (Oteiza et al. 2005; Pawlikowska-Pawlęga et al. 2012). The presented results obtained by means of fluorimetric and ^1^H-NMR techniques, on the dynamic and structural properties of lipid membranes in their interaction with active compounds like flavonoids and phenolic acids, indicate the existence of a close connection between adsorption (intercalation) of components of the JQ extract in the membranes and JQ effectiveness to protect them against attacks by free radicals.

The mechanisms of action of many phenolic compounds are thought to be via their free-radical scavenging activities or the inhibition of pro-inflammatory enzymes in inflammatory cascades (Sadik et al. 2003). The potential anti-inflammatory capacity of JQ extract was shown on the basis of the inhibition of enzymes of the cyclooxygenase group (COX-1 and COX-2). COX-1 catalyzes the production of prostaglandins involved in digestive tract mucosal protection and other physiological activities, whereas COX-2, especially inducible, is responsible for the production of prostaglandins that mediate inflammation, pain, and fever (Tacca et al. 2002). Our studies have shown that JQ extract has the capacity to inhibit the enzymes COX-1 and COX-2 (Table 4). The literature reports confirm the anti-inflammatory capacity of *C. speciosa*, determined on an animal model by Li and co-workers, where a 10 % ethanol fraction of *C. speciosa* had stronger anti-inflammatory effects, which was evaluated using carrageenan-induced paw edema in rats (Li et al. 2009). Our earlier studies showed that JQ extract caused approximately 18 % inhibition of LOX-1 at a concentration of 8 µg mL^−1^ (Strugała and Gabrielska 2014). From our research using nonsteroidal anti-inflammatory drugs (NSAIDs), indomethacin, and ibuprofen, it follows that they cause inhibition of inflammatory enzymes at concentrations much lower than JQ extract (Table 4; Strugała and Gabrielska 2014). However, despite the significant therapeutic efficacy, NSAIDs can cause side effects, in particular in relation to the digestive system and kidneys (Cronstein 2002). This process is associated with the unfavorable inhibition of COX-1 activity, which is why the selective inhibition of COX-2 is more desirable. Here, the results gave a COX-2/COX-1 selectivity ratio of 0.48 determined on the basis of the values of IC_50_ for JQ, which shows that the tested extract shows more than twofold higher activity toward COX-2 than COX-1.

Determination of the degree of binding of components of the extract/potential drug with albumin is one of the basic factors for the healthy properties of the extract. From the literature reports, it is known that deposition, transportation, metabolism, and efficacy of drugs are strongly affected by their binding to HSA (Bourassa et al. 2011; Ji et al. 2002). These important functions performed by albumin in the body turned our attention to the need to examine the interactions of albumin and JQ extract and its components. Effects of JQ extract and EC and CA on HSA were examined by analyzing changes in the intensity of the natural fluorescence of the protein. The concentration-dependent changes in HSA fluorescence intensity testify to the associations of JQ and its components EC and CA with albumin (Fig. 4). The binding constant shows the power of the ligand–protein associations and thus can be used for comparison of the binding affinities of structurally related ligands to a protein molecule. From our studies, it follows that the Stern–Volmer constant for the JQ extract–albumin association is 43.4 × 10^3^ mL g^−1^. Its value for (−)-epicatechin (3.7 × 10^3^ M^−1^) is by an order of magnitude lower than the constant for binding of chlorogenic acid (3.0 × 10^4^ M^−1^). The binding constant we determined for CA is in accordance with those reported by Hu et al. (2012) and Sinisi et al. (2015), which is at the level of 10^4^ M^−1^. There are numerous reports showing the quenching of intrinsic fluorescence of HSA upon interacting with various drug molecules. For instance, Wilgusz and co-workers found that the third-generation drug meloxicam binds with plasma protein at 99 % with an association constant of the order of 10^5^ M^−1^, (Wilgusz and Trynda-Lemiesz 2014). In addition, usually drugs bind to high-affinity sites with typical association constants in the range of 10^4^–10^6^ M^−1^. It can therefore be stated that the natural substances studied in this work show a relatively high affinity to human albumin. The quenching mechanism of albumin is classified as either static or dynamic quenching (Lakowicz 2006). The calculated values of the bimolecular quenching rate constant (*K*_*Q*_), which reflects quenching or accessibility of the fluorophore to a quencher, for EC and CA are 7.40 × 10^11^ and 6.04 × 10^12^ M s^−1^, respectively (for JQ it is 8.68 × 10^11^ mL g^−1^ s^−1^). In order to specify the static mechanism of quenching, one of the criteria is the constant *K*_Q_, which is greater than the diffusion-limited rate constant of the biomolecule, equal to 1.0 × 10^10^ M^−1^ s^−1^ (Lakowicz 2006). The values of *K*_*Q*_, obtained in our work, for the studied compounds may suggest that the static quenching mechanism is the main reason for albumin fluorescence quenching, which is in accord with studies of other authors (Trnková et al. 2011; Ji et al. 2002).

Our study clearly demonstrated that JQ extract can promote the growth of *L. casei* and *L. plantarum* (Fig. 5). The stimulating influence of phenolics on the growth of probiotic bacteria has been discussed in a number of papers. Yang and co-workers found that 10 % blackberry juice showed positive effects on the growth of *L. casei* and *L. plantarum* (1–4 log CFU mL^−1^) (Yang et al. 2014), whereas other researchers have shown that epicatechin in 44.86–12.19 nmol g^−1^ concentration practically does not stimulate or inhibit the growth of *L. casei* and *Lactobacillus rhamnosus* (Lee et al. 2006). The stimulating influence of JQ extract, found in the present work, on bacteria of the *Lactobacillus* family may be due to the unique composition of compounds, including phenolics, and the probability that certain extract compounds may act in combination and have a synergistic effect on the growth of *Lactobacillus* bacteria (Goto et al. 1998). A more plausible explanation for the stimulatory effect of JQ on bacterial growth is that some microorganisms are able to use these compounds as substrates. Certain members of *Lactobacillus* possess the ability to metabolize phenolic compounds during growth, and therefore phenols would be supplying energy to the cell.

CF, as a hydrophilic fluorescence marker, is useful in structural studies of lipid vesicles. CF released from liposomes shows measurable fluorescence. By tracing the release induced by changes in the liposome membrane, one can estimate the action of an agent which affects the membrane. Based on the studies of other authors, it follows that CF and calcein permeation rates across the membranes correlate with membrane fluidity (Shimanouchi et al. 2009). The PC liposome membrane at room temperature is in the liquid-crystalline state, demonstrating high disorder (fluidity) of the hydrocarbon chains in the lipid bilayer. For this reason, CF permeation across the hydrophobic zone with high fluidity is relatively easy. Paradoxically, the presence of JQ molecules, and its components used in the CF leakage test, that accumulate to a large extent in the polar, surface area of the membrane of PC liposomes, cause a small (within 3–11 %—Fig. 6) increase in the leakage. One can assume that the incorporation of molecules of extract into the outer layer of the membrane causes local structural defects in the membrane, which foster the diffusion of CF molecules through the membrane.

## Conclusion

To sum up, it can be stated that *C. speciosa* fruit ethanolic extract has broad biological activity. Its antiradical and antioxidant activities effectively protect the lipid and erythrocyte membranes against oxidation induced by physicochemical factors. The extract also inhibits activities of the pro-inflammatory enzymes, COX-1 and COX-2, exhibiting over two times greater affinity for COX-2 than that for COX-1. JQ extract has a high affinity for binding with albumin, the main protein of the human blood plasma, which is the basic factor determining its bioavailability. *Chaenomeles speciosa* extract has a potential to be used as a promising natural product, as well as a growth promoter for beneficial bacteria, specifically *Lactobacillus*. This may have an important influence on the physiology and biochemistry of the gut. The results of the biophysical studies presented in this paper allow us to conclude that JQ extract components can interact mainly with the lipid polar head groups at the lipid–water interface of membranes and protect the lipid bilayer against aggression by deleterious molecules. If the damaging molecule is an oxidant, this protective effect could contribute to the overall antioxidant action of JQ extract. Lipid membrane rigidification can hinder the diffusion of free radicals, reduce the kinetics of oxidative reactions, and inhibit the propagation of lipid peroxidation, e.g., caused by UVB radiation and free radicals in the form of AAPH^·^ molecules. The abilities of JQ extract and its components to interact with lipid and lipid–protein membranes are of vital biologic and medical importance, which is connected with the application of the extract as an antioxidant and a drug that boosts the therapy of many illnesses caused by inflammatory processes and oxidative stress.
